# The Effect of Cement Space Parameters on the Marginal Adaptation of Milled Endocrowns: An In Vitro Study

**DOI:** 10.7759/cureus.38688

**Published:** 2023-05-07

**Authors:** Oubada Suliman, Mohammad R Rayyan

**Affiliations:** 1 Prosthodontic Department, Riyadh Elm University, Riyadh, SAU

**Keywords:** marginal adaptation, lithium disilicate, endocrown, cement space, cad-cam

## Abstract

Background

Cement film space plays a significant role in achieving good adaptation of indirect restorations. The objective of this study is to investigate the effect of cement space parameters on the marginal adaptation of computer-aided design (CAD)/computer-assisted manufacturing (CAM) endocrowns.

Methodology

The coronal part of 10 freshly extracted human mandibular molars was reduced to a level of 1.5 mm above the cementoenamel junction (CEJ); then, root canal treatment was performed. On each tooth, four lithium disilicate endocrowns with different cement space parameters (40, 80, 120, and 160 µm) were designed and fabricated using CAD/CAM. Endocrowns were seated to their prepared teeth, and the vertical marginal gap was measured in 20 equidistant points for each endocrown using a stereomicroscope on 90× magnification. The mean marginal gaps of the four groups were compared using a one-way analysis of variance (ANOVA) and the Tukey honestly significant difference (HSD) test considering p<0.05 as the cutoff for statistical significance.

Results

The mean marginal gap of the 40 µm, 80 µm, 120 µm, and 160 µm groups were 46.25±21.20 µm, 21.75±11.10 µm, 15.94±06.62 µm, and 13.10±07.08 µm, respectively. One-way ANOVA showed a significant difference in the marginal gaps between groups (p<0.001). The Tukey post hoc test showed a statistically significant mean difference between 40 µm and each of the other three groups (p<0.001).

Conclusion

The cement space parameter variation affects the marginal adaptation of endocrowns. The cement space of 40 µm resulted in a higher marginal gap than cement spaces of 80, 120, and 160 µm.

## Introduction

Endodontically treated teeth are frequently associated with significant tooth loss and thus often require more comprehensive restorative treatment to achieve adequate function, aesthetics, and preservation of the remaining tooth structure [1]. One such treatment option is an endocrown, which was introduced by Pissis [2] in 1995 as a solution for extensively damaged endodontically treated molars. Endocrowns are designed to engage the pulp chamber of the tooth for maximum retention, eliminating the need for additional retention features such as posts and cores, thus reducing treatment cost and time [2,3]. The choice of ceramic material for the endocrown restoration is critical to its success since the concept of this restoration requires an all-ceramic material that can be etched and bonded to the tooth structure. Additionally, it should have adequate mechanical properties suitable for use in the posterior teeth. Lithium disilicate is considered an optimum material for endocrowns due to its excellent mechanical and optical properties. Its flexural strength is adequate for use in the posterior teeth, and it can be acid-etched and bonded, which made it a popular choice for single-tooth all-ceramic restorations [3].

One of the critical factors in providing a long-lasting restoration is maintaining minimal marginal and internal discrepancies, as it helps to preserve the tooth and surrounding periodontium health [4]. Inadequate restoration margins are susceptible to microleakage and may result in secondary caries, which is one of the most common complications of single-tooth restorations [5,6].

There are several factors reported in the literature that influence the size of the marginal gap, including cement thickness, cement type, tooth type, finish line configuration, tooth preparation, restoration material type, restoration fabrication method, and impression type [7-11].

The interface between the restoration and prepared tooth structure plays a significant role in achieving good adaptation and is referred to as the cement film space thickness [12]. Adequate space for cement can be preserved by using a die spacer during conventional fabrication or by adjusting the parameters of computer-aided design (CAD)/computer-assisted manufacturing (CAM) software, which allows for the relief of the internal surface of the restoration [13,14].

Published studies on CAD/CAM restorations have investigated various properties and materials, but there is no agreement on the optimal cement space parameter for a specific type of restoration [15,16]. CAD/CAM software usually provides recommendations for cement spacer parameters for different types of restorations, regardless of the material used for fabrication. For example, CEREC recommends a 100 µm cement space for inlays and onlays and 120 µm for single crowns [4].

Several studies have investigated the effect of cement space thickness on the marginal integrity of conventional crowns, with some concluding that a cement space parameter of 30-60 µm provides better adaptation while others recommend higher values ranging from 80 to 150 µm for improved marginal adaptation [17-20]. However, there is a lack of similar studies on endocrowns, which motivated this in vitro study to investigate the effect of cement space parameters on the marginal adaptation of lithium disilicate endocrowns fabricated with CAD/CAM. The study's null hypothesis was that cement space parameters have no effect on the marginal adaptation of lithium disilicate endocrowns.

## Materials and methods

The study proposal was registered in the Research Center of Riyadh Elm University and approved by the Institutional Review Board (IRB) of Riyadh Elm University (approval number: RC/IRB/2019/73).

This was an in vitro controlled trial study in which four groups of lithium disilicate endocrown restorations (n=10) were fabricated using different cement space parameters. Ten freshly extracted human mandibular molars were selected for this purpose, and for each tooth, four identical endocrowns were fabricated with the only difference in cement space parameter to create four groups (n=10) (Table 1). This sample size will achieve a power of 0.8, assuming an effect size of 0.55 and an alpha error of 0.05.

**Table 1:** Study groups

Group	Sample size	Axial and occlusal cement space
G40 µm	10	40 µm
G80 µm	10	80 µm
G120 µm	10	120 µm
G160 µm	10	160 µm

A total of 10 freshly extracted human permanent mandibular molars were selected. The extracted teeth were cleaned with an ultrasonic scaler and stored in 10% formalin. The included teeth were caries-free with no cracks or fractures at least 2 mm above the cementoenamel junction (CEJ), and the pulp chamber depth was at least 3 mm. The teeth with caries, cracks, or fractures extending less than 2 mm to the CEJ and the teeth with a pulp chamber depth of less than 3 mm were excluded. The teeth were sectioned at a level of 1.5 mm above the highest level of CEJ parallel to the occlusal plane using a diamond precision saw (ISOMET 2000 Precision Saw, Buehler, Leinfelden-Echterdingen, Germany).

All teeth were endodontically treated by one experienced endodontist. Access cavity preparation and a complete de-roofing of the pulp chambers using low-speed handpiece with rounded carbide bur were done. Then, canal orifices were shaped using Gates Glidden drills (X-Gates, Dentsply Sirona, Bensheim, Germany). The working length was determined on a radiograph using a size 10 K file (SybronEndo, Kerr Endodontics, Gilbert, AZ). Canals were then manually instrumented with K files up to size 20; then, cleaning and shaping were completed using nickel-titanium (NiTi) rotary system (ProTaper Gold, Dentsply Sirona, Bensheim, Germany) with normal saline and sodium hypochlorite (2.5% NaOCl) irrigation. Canals were dried with Absorbent Paper Points (Meta Biomed, Cheongju, South Korea) and then obturated using ProTaper Conform Fit gutta-percha points (ProTaper Gold Gutta Percha, Dentsply Sirona, Bensheim, Germany) and sealer (AH-Plus, Dentsply Maillefer, Tulsa, OK) using lateral condensation technique. After obturation, excess gutta-percha was removed, and the pulp chamber was cleaned.

**Figure 1:**
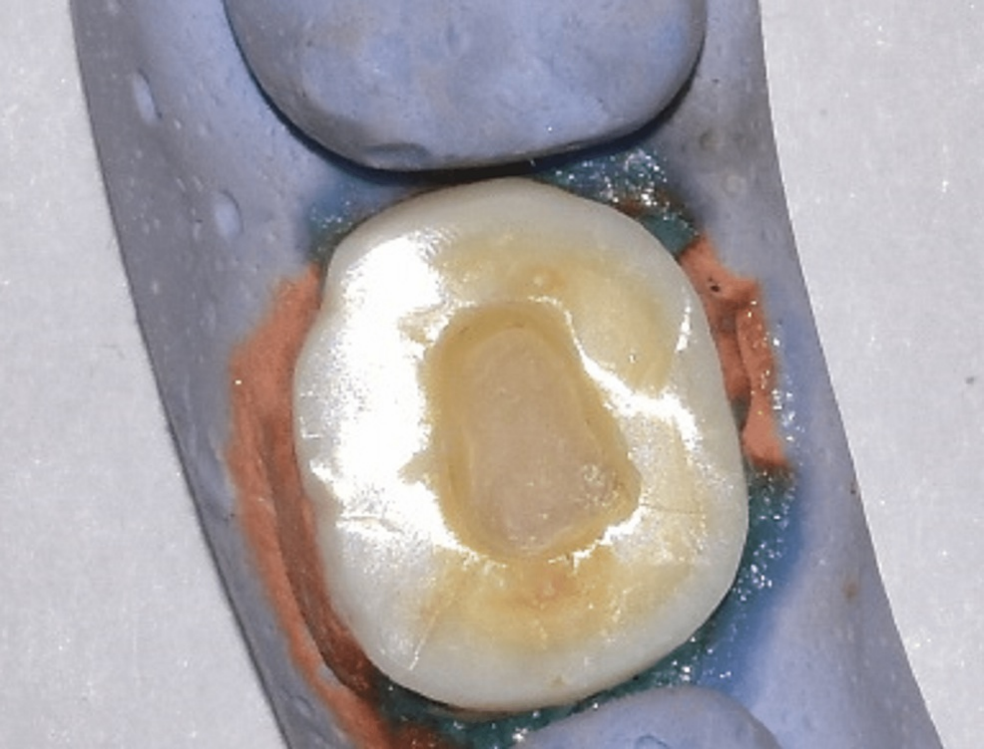
Occlusal view of a sample tooth after preparation for endocrown

After endodontic treatment, orifices and the pulp chamber floor were covered with bulk-fill composite resin (Filtek, 3M, Saint Paul, MN) to achieve a uniform pulp chamber depth of 3 mm for all teeth. Cavity preparation was limited to the removal of undercut areas within the pulp chamber using a fine round-end taper diamond (8856.31.016 FG, Brasseler, Savannah, GA) and achieving 8° occlusal divergence for the inner walls. Butt joint margins and cavosurface angles were finished using fine finishing disks (Sof-Lex, 3M, Saint Paul, MN) (Figure 1).

A dentoform (Columbia Dentoform, Lancaster, PA) was duplicated to obtain standardized 10 mandibular casts and one maxillary stone cast. In the mandibular casts, the right first molars were trimmed out to allow the placement of the sample teeth. Each tooth was positioned in one cast with buccal butt joint margin at a level of 1.5 mm above the buccal gingival margin of the adjacent teeth.

**Figure 2:**
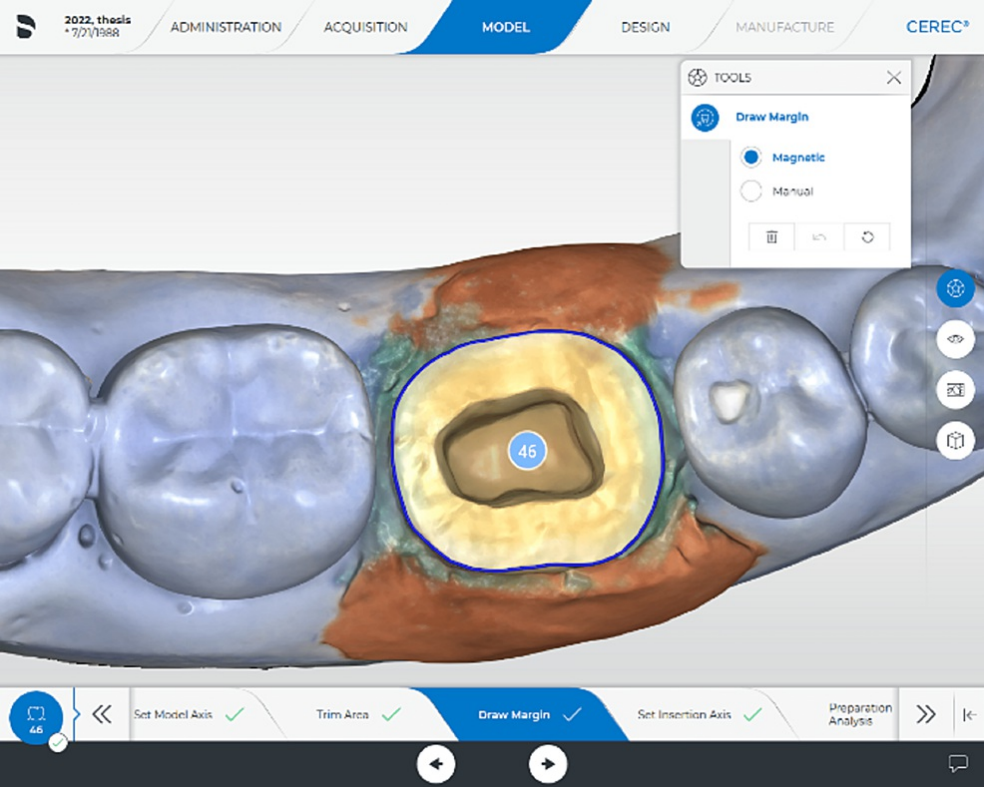
Digital impression of the mounted sample using intraoral scanner

After mounting, digital impressions were obtained using powder-free intraoral scanner (CEREC Omnicam, Sirona Dental Systems GmbH, Bensheim, Germany) (Figure 2).

Endocrown restorations were designed using CAD software (InLab software version 5.2.8, Dentsply Sirona, Bensheim, Germany). Each design was duplicated four times and saved as four separate files. The biogeneric design was applied for all the restorations with all default parameters recommended by the software except for the digital cement spacer that was adjusted for each of the four duplicates of each specimen as 40, 80, 120, and 160 µm.

Endocrowns were produced using lithium disilicate glass-ceramic material blocks (IPS e.max CAD, Ivoclar Vivadent, Schaan, Liechtenstein), which were milled using CEREC InLab MCXL milling unit (Dentsply Sirona, Bensheim, Germany). The crystallization of lithium disilicate restorations was done using Programat P310 furnace (Ivoclar Vivadent, Schaan, Liechtenstein), according to the device's preprogrammed software settings.

For marginal adaptation, an evaluation of the absolute distance between the external margin of the restoration to the finish line was done using a 3.5× to 180× trinocular zoom stereomicroscope (SM-3TZZ-54S-14M-B, AmScope, Irvine, CA) attached with a 14 MP digital camera (MU1403, AmScope, Irvine, CA) at 90× magnification.

**Figure 3:**
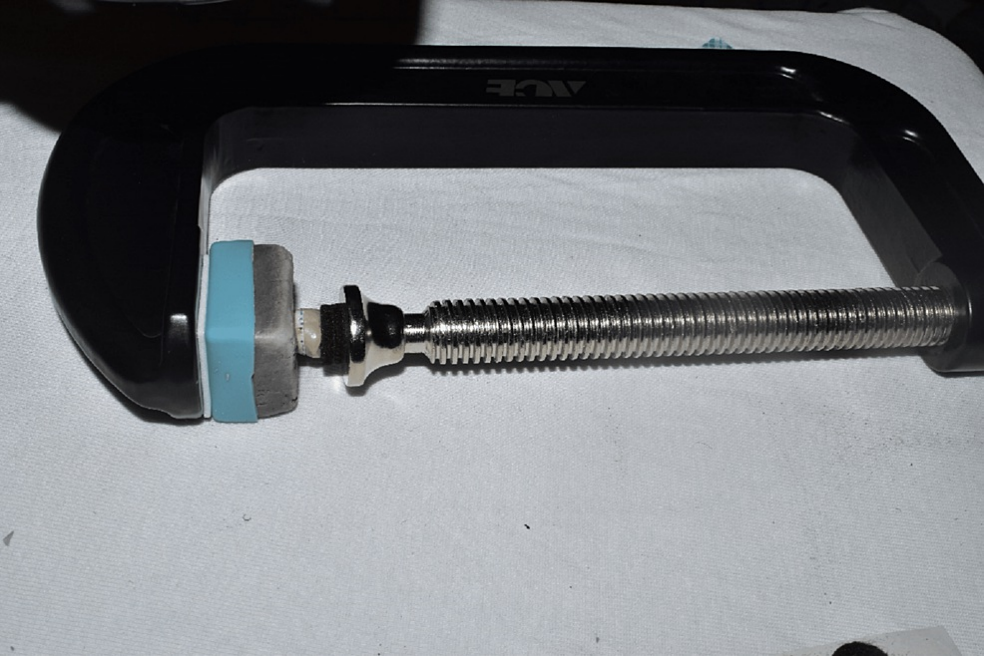
Holding device with customized base to standardize the position of samples under the stereomicroscope

The roots of the sample teeth were embedded in self-cured acrylic resin (DeguDent, Hanau, Germany) parallel to their long axes, in a standardized square-shaped mounting mold. For the purpose of standardized specimen positioning for stereomicroscopic examination, a customized base was fixed to a universal holding device that allows the positioning of all samples in the same position under a stereomicroscope (Figure 3).

**Figure 4:**
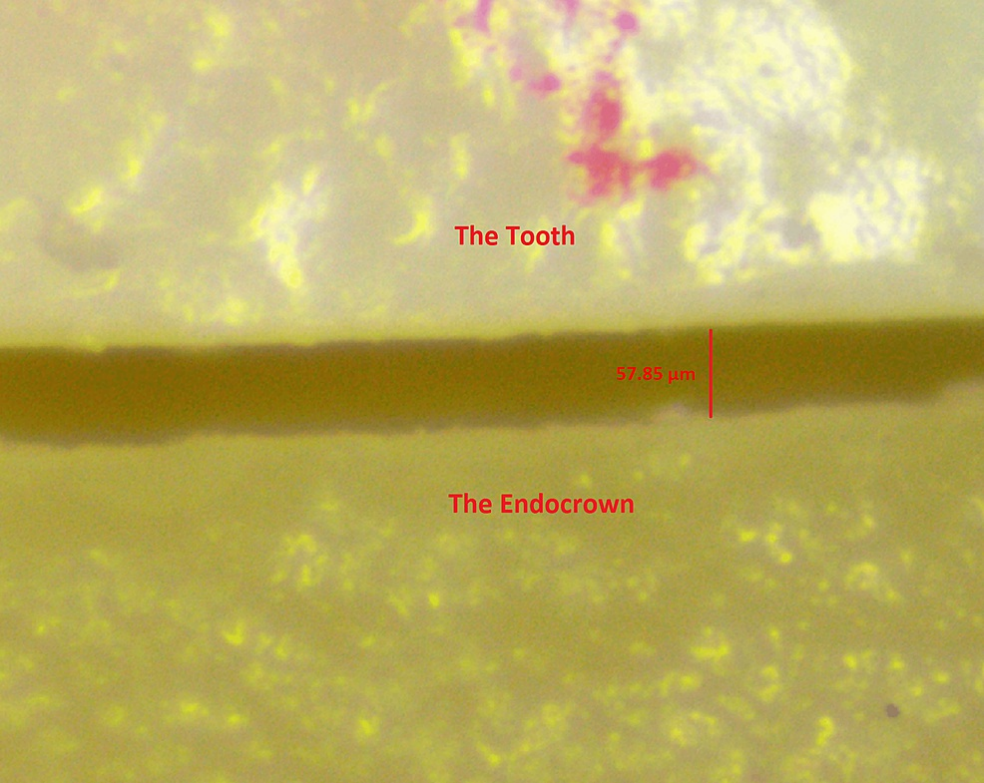
Measurement of the marginal gap at the standardized points

The camera was calibrated by a precise stage calibration slide (MR095, AmScope, Irvine, CA) with a total length of 1 mm subdivided into 100 divisions of 0.01 mm each, under a magnification of 90×. Twenty measuring points were marked as five equidistant points on each of the four surfaces of the sample teeth, points were marked using a 0.1 marker pen around 0.5 mm just below the butt joint margin, and measurements were made in alignment with one border of the marking at each point (Figure 4).

For each sample tooth, measurements were repeated for each one of the four endocrowns with the four different cement spaces at the exact same points. All measurements were made in a real-time video stream with 4096×3286 resolution on a computer using the AmScope software (Amscope, X64, 4.11.201313.20220108, AmScope, Irvine, CA).

The vertical marginal gaps in the 20 points for each endocrown were recorded on an Excel sheet (Microsoft® Corp., Redmond, WA). Data were analyzed using the Statistical Package for Social Sciences (SPSS) version 26 (IBM SPSS Statistics, Armonk, NY). Descriptive statistics were presented using mean, standard deviation, minimum, and maximum. The comparison between the mean values of marginal gaps among each group had been conducted using the one-way analysis of variance (ANOVA) test. Post hoc analyses were performed using the Tukey honestly significant difference (HSD) test. Two-tailed analysis with p<0.05 was used as the cutoff for statistical significance.

## Results

The descriptive statistics of the study variables are given in Table 1. It was revealed that the mean value of marginal gaps was highest at G40 µm (46.25±21.20 µm), followed by G80 µm (21.75±11.10 µm) and G120 µm (15.94±06.62 µm) while it was lowest at G160 µm (13.10±07.08 µm) (Table 2).

**Table 2:** Marginal gap of the study samples SD, standard deviation

Variable group	Mean (µm)	SD (µm)	Minimum (µm)	Maximum (µm)
G40 µm	46.25	21.20	21.40	91.25
G80 µm	21.75	11.10	10.75	46.85
G120 µm	15.94	06.62	09.15	30.70
G160 µm	13.10	07.08	02.30	27.65

Using the one-way ANOVA test (parametric test), there was a significant difference in the mean marginal gap values between the four groups (p<0.001).

In post hoc analysis, it was revealed that significant differences were observed between G40 µm and G80 µm (p=0.001), G120 µm (p<0.001), and G160 µm (p<0.001) while there were no statistically significant differences between the G80 µm, G120 µm, and G160 µm groups (Table 3).

**Table 3:** Post hoc test to determine the multiple mean differences of marginal gaps in the study groups *The mean difference is significant at the 0.05 level

Multiple comparisons						
Groups (I)	Groups (J)	Mean difference (I-J)	Standard error	Significant	95% confidence interval	
					Lower bound	Upper bound
G40 µm	G80 µm	24.50000^*^	5.77326	0.001*	8.9513	40.0487
	G120 µm	30.31000^*^	5.77326	0.000*	14.7613	45.8587
	G160 µm	33.15500^*^	5.77326	0.000*	17.6063	48.7037
G80 µm	G40 µm	-24.50000^*^	5.77326	0.001*	-40.0487	-8.9513
	G120 µm	5.81000	5.77326	0.747	-9.7387	21.3587
	G160 µm	8.65500	5.77326	0.449	-6.8937	24.2037
G120 µm	G40 µm	-30.31000^*^	5.77326	0.000*	-45.8587	-14.7613
	G80 µm	-5.81000	5.77326	0.747	-21.3587	9.7387
	G160 µm	2.84500	5.77326	0.960	-12.7037	18.3937
G160 µm	G40 µm	-33.15500^*^	5.77326	0.000*	-48.7037	-17.6063
	G80 µm	-8.65500	5.77326	0.449	-24.2037	6.8937
	G120 µm	-2.84500	5.77326	0.960	-18.3937	12.7037

Three of the samples in G40 µm had marginal gaps above 120 µm, while none of the samples in the other groups exceeded the 120 µm marginal gap threshold.

## Discussion

The aim of this study was to investigate the effect of cement space parameters on the marginal adaptation of lithium disilicate endocrowns fabricated using CAD/CAM technology. The marginal fit and intimate adaptation of the restoration to the tooth is crucial for the success and survival of indirect restorations [4]. The effect of cement space thickness on the marginal adaptation of conventional crowns has been a topic of interest in previous studies [14-20], but to the best of the authors' knowledge, no study has investigated this effect on endocrowns.

In the current study, the marginal adaptation was measured in terms of the vertical marginal discrepancy, which has been used in various in vitro studies. The vertical marginal discrepancy was measured using a direct method with a stereomicroscope, which is a simple and conservative method that avoids procedural errors that may result from other techniques such as sectioning and replication [12]. To standardize measurement points in the current study, 40 endocrowns were fabricated using 10 sample teeth and four endocrowns per tooth according to the cement space value for each group, with a uniform distribution of measuring points. The cement space thicknesses used for the study groups were 40, 80, 120, and 160 µm, with 120 µm recommended as the default by the CEREC software and the other values selected as equidistant values to cover the commonly reported range in literature.

The results of the study showed that the marginal gap mean values ranged from 13.10 µm to 46.25 µm, which are below the clinically acceptable value of 120 µm as proposed by McLean and von Fraunhofer [21]. These results are consistent with previously reported values for endocrowns [8,9]. However, it should be noted that while the mean values in all study groups were below the acceptable limit, 10 out of 200 points in the 40 µm group had measurement values above 120 µm, indicating the importance of careful consideration of cement space parameters. Reducing the cement space thickness could increase the chance of interferences in the CAD/CAM restorations, which will affect their marginal fit [20].

The study found that there was a significant difference in mean marginal gaps between the study groups, so the null hypothesis was rejected. The increase in cement space thickness to 80 µm and above led to smaller marginal gap values, which is in agreement with the findings of previous studies on conventional crowns. Mously et al. [20] reported a higher marginal gap when 30 µm cement space thickness was used in contrast to 60 µm or 100 µm. Similarly, Hmaidouch et al. [19] reported a higher marginal gap in copings with 50 µm cement space thickness than the ones fabricated with 100 µm cement space parameter. The results of the current study and the previous studies indicate that higher cement space thickness could accommodate a higher margin of fabrication error and improve the adaptation of the restoration while a smaller cement space could affect the seating of the restoration and result in higher marginal discrepancy [19,20]. A study by Kale et al. reported a significant increase in the marginal discrepancy of monolithic zirconia crowns when the cement space was decreased from 50 µm to 40 µm and 30 µm, which indicates that even a small difference (10 µm) in cement space thickness affects the marginal fit of restorations [14].

In contrast to our findings, Dauti et al. [17] studied the marginal adaptation of polymer-infiltrated ceramic network crowns and reported no significant influence of cement space thickness on the marginal fit. They have compared only two values of cement space (50 µm and 80 µm). Similarly, Shim et al. [18] reported no difference in marginal adaptation when 40 µm or 80 µm cement space parameter was used. The conflict with our study's results could be contributed to the difference in restoration design, material, and CAD/CAM system [17,18].

There are limitations of this study that have to be reported. One of them was the use of natural extracted human teeth, which are difficult to standardize. However, natural teeth are closer to resembling the clinical situation regarding the architecture and contours of the pulp chamber, which is critical in endocrown concept. Also, marginal gaps were measured without the cementation of the endocrowns in the current study. Cementation could affect the marginal gap, and this must be taken into consideration when interpreting the results of the current study [9,13,17].

## Conclusions

Applying adequate cement space thickness is crucial for the adaptation and seating of restorations. Within the limitation of this in vitro study, it was concluded that the cement space parameters affect the marginal adaptation of CAD/CAM endocrowns. The cement space thickness of 40 µm was accompanied by an increase in marginal gap compared to 80, 120, and 160 µm. Cement space parameters less than those recommended by the manufacturers could negatively affect the fitting of CAD/CAM endocrowns and increase the chance of open margins.
